# Sleep medicine in Taiwan

**DOI:** 10.1007/s41105-015-0007-9

**Published:** 2015-11-18

**Authors:** Ning-Hung Chen, Liang-Wen Hang, Chia-Mo Lin

**Affiliations:** Sleep Center, Department of Pulmonary and Critical Care Medicine, Chang Gung Memorial Hospital, 5, Fu-Shin Street, Kweishan Shang, Taoyuan, 333 Taiwan; Department of Respiratory Therapy, Chang Gung University, Taoyuan, Taiwan; Sleep Medicine Center, Department of Internal Medicine, China Medical University Hospital, Taichung, 40402 Taiwan; Department of Respiratory Therapy, College of Health Care, China Medical University, Taichung, Taiwan; Sleep Center, Pulmonary and Critical Care Medicine, Shin-Kong Wu Ho-Su Memorial Hospital, Taipei, Taiwan; Medical College, Fu Jen Catholic University, Taipei, Taiwan

**Keywords:** Sleep medicine, Taiwan, Sleep education, Sleep research

## Abstract

The sleep medicine is a young medical science in Taiwan. It began from less than 10 sleep beds 20 years ago in four hospitals all over Taiwan. By the organization of sleep team in Chang Gung Memorial Hospital and the initiation of Taiwan Society of Sleep Medicine, sleep medicine becomes a popular medicine in the past decades. The setting of Sleep Society in 2002 is the milestone to promote the sleep medicine, educate the public and professionals, and control of the quality of clinical practice. Epidemiologic study in Taiwan shows many Taiwanese suffer from sleep disorders and hence more sleep institutes are needed. Accreditation has become a mission of the Taiwan Society of Sleep Medicine. Technicians, sleep centers, sleep specialists and sleep phycologists are gradually certified by the society. 215 sleep technicians, 307 sleep physicians, 31 iCBT therapists and 21 sleep centers are certified by the society till 2015. The first sleep related medical courses are initiated in the Department of Respiratory Therapy in Chang Gung University from 2003. For the following years, eight medical courses are set in six Universities now. Given the fact that the Asian accounts for the largest proportion of population in the world, investigation on the OSA in Asian population is essential. In this article, we aimed to demonstrate the outcomes of OSA-related research in Asia. In particular, the progress driven by the studies in Taiwan will be discussed. Data were obtained online from the Science Citation Index Expanded database of the Thomson Reuters’ Web of Science Core Collection. Keywords including “apnea” and “hyponea” were used to search by applying the filters of the title and the publication years between 1991 and 2014. In total, 2623 articles were hit, subject to the criteria for data search. Among the 2623 articles, sleep and breathing related articles (128, 4.95 %) were the most frequently reported. Japan is the country that published the highest amount of OSA-related articles. The Asian institutions that ranked the first two in the number of OSA-related articles were Technion-Israel Institute of Technology and Tel Aviv University in Israel. In Taiwan, Chang Gung Memorial Hospital and Chang Gung University ranked fourth and fifth. Both institutes reported 63 articles. In Asia, Japan leads in the quantity of publication and the Japanese research institutes performed evenly. China had rapid growth in the number of articles since 2011. Although sleep medicine developed smoothly in the past decades in Taiwan, there were problems that the sleep society and specialists had to encounter. Insurance limits the expansion of sleep labs and the reimbursement is very low for sleep medicine to survive. The affiliations of sleep specialist and the sleep education are also important issue that the sleep specialists in the society have to discuss.The previous achievements do not guarantee future success. We have to face these problems seriously and take action for the following years to maintain the development of sleep medicine in Taiwan.

## Introduction

### History and position

Sleep medicine is one of the latest developed specialties in western medicine in Taiwan. Although sleep problem is recognized long time ago in Chinese Medicine, in western medical development, sleep medicine never became a well-developed specialty in the health care system in Taiwan. The first sleep lab in Taiwan was set up in Mackay Memorial Hospital 25 years ago. One to two bed rooms for overnight polysomnography (PSG) were set up for patients suspected to have obstructive sleep apnea. Most of the technicians at that time were affiliated to the laboratory of pulmonary department. They have to work for pulmonary function test at day time and PSG on the night shift. There was a joke from the patients that the snore of the technician is louder than the patients during those times. There were four sleep labs over Taiwan which belong to Mackay Memorial Hospital, Veteran General Hospital, Chang Gung Memorial Hospital and Taipei City Psychiatry Center. Less than 10 PSG beds were serviced for 23 million Taiwanese before 2000.

### Multi-disciplinary clinics, sleep specialty and sleep medicine course were first set up in Chang Gung Memorial Hospital since 1994 in Taiwan

By the introduction of laser-assisted uvulopharyngoplasty (LAUP) in Taiwan in 1980, the otolaryngologists were interested on the patients of snore and obstructive sleep apnea syndrome. The number of patients increased quickly and the waiting list for polysomnography got longer rapidly. The superintendent of Chang Gung Memorial Hospital (CGMH) decided to expand the sleep beds and appointed full-time technicians for PSG. This is the first full-time technician for PSG in Taiwan. Sleep research team including pulmonologist, otolaryngologist, plastic surgeon, orthodontics, radiologist, and pediatricians were organized in Chang Gung Memorial Hospital since 1996. Multi-disciplinary clinics for patients of sleep apnea were also set up in CGMH soon after. This is also the first sleep clinics in Taiwan with multi-disciplinary approaching for patients. For the following years after 2000, the members of sleep team in CGMH went overseas for sleep specialist training in Stanford Sleep Disorders Clinics in USA with Professor Guillenminault and Professor Kushida, Edinburg Sleep Center in England with Professor Neil Douglas, Shiga University in Japan with Professor Okawa, Toronto University in Canada with Professor Bradley and University of Pennsylvania with Professor Schwab and Professor Pack. The first educational course of sleep medicine was set up in the Chang Gung University for medical students since 2003. Certification of sleep specialty was conducted in CGMH for members since 2006.

### The Taiwan Society of Sleep Medicine (TSSM) was set up on 21 March 2002

In November 2nd 2001, Dr. Kwan‐Ming Shao, who is the director of sleep labs in Veteran General Hospital, initiated the preparing committee of Taiwan Society of Sleep Medicine including physicians, researchers and technicians on sleep medicine. On 18 June 2001, the committee applied for the formal society to the government and got approval on 19 Oct. The first members conference took place on 23 March 2002 with 276 members include physicians from every disciplinary, technicians, psychologists and researchers. The missions of the society are promotion, education, accreditation and research (Table 1). The setting up of the society is the milestone of the development of sleep medicine in Taiwan (http://www.tssm.org.tw/about.php?key=about01). Before the society, sleep medicine was practiced in different disciplinary by a minority of physicians. The Sleep Society provides a platform for every disciplinary to discuss about the sleep medicine. It empowers the promotion of sleep medicine on clinical medicine, research and public awareness. Professor Hsiao who is a pulmonologist became the first president of TSSM and then Professor Li who is a psychiatric in National Taiwan University Hospital was elected as the second president. Multiple disciplinary involvements were empathized in this society. By the organization of the society, sleep medicine could be developed smoothly in Taiwan.

**Table 1:** Mission of Taiwan society of sleep medicine

Promotion	Promotion of sleep medicine in professional and public	
	Professional	Lecture and seminar in the Hospital
	Public	Public health issue in media press every March accompany with the annual conference
		Media and newspaper
		Website
Education	Educate the professional and public of sleep medicine	
	Annual conference	Annual conference of Taiwan Society of Sleep Medicine every March
	Symposium	Symposium on specific topic of sleep disorders
	Training course	Regular training course every year for sleep technicians, sleep specialist, and sleep psychologist
Accreditation	Set up and conduct the certification of sleep specialty in all disciplines	
	Sleep technicians	Certification examination since 2006
	Sleep lab	Since 2009
	Sleep specialist	Since 2012
	Sleep psychologist	Since 2012
Research		
	Platform for research between industry and sleep professionals	Initiate epidemiology study in Taiwan

### Accreditation of sleep technicians, centers, physicians, and iCBT therapists

Taiwan Society of Sleep Medicine (TSSM) currently has around 900 active members, including 400 physicians, 150 technicians, 50 psychologists, and 300 sleep-related professional persons.

Currently Taiwan Sleep Society gives yearly certification for sleep laboratories, physicians, insomnia behavior therapists, and technicians (Table 2).

**Table 2:** Certifications of Taiwan Society of Sleep Medicine till 2015

Certification	Year begin	Number of sessions	Number of qualifiers
Sleep technician	2007	11	215
Sleep physicians	2011	6	307
CBTi therapists	2012	4	31
Sleep centers	2009	8	21
			4 September, 2015

To improve the quality of sleep polysomnography, TSSM started to authenticate 215 sleep technicians since 2007. To standardize the set up of sleep laboratory, TSSM had started sleep laboratory accreditation since 2009. 21 sleep laboratories now own the accreditation. As the most difficult part in certification process, the board committee TSSM has started accreditation for physician from late 2011. 307 sleep physicians had been certified as sleep specialties till 2015. To qualify more insomnia cognitive behavior therapists, TSSM started the accreditation process from 2012. All these accreditation procedures included the following essential requirement: (1) the attendee must be a medical related profession and have license for practice. (2) The attendee must be a member of TSSM and have 40 h of CME before certification. (3) The attendee must attend the core education course organized from TSSM specifically for certification procedure that includes essential issues in sleep medicine, such as sleep physiology, sleep pharmacology, insomnia, OSA, movement disorders, pediatric sleep disorders, etc. Technical course is also required for technicians of therapists. (4) The attendee to have a 1-year experience in clinical jobs that related to sleep disorders before certification. (5) Finally, the attendee has to pass an examination for certification. Persons who fulfill all these criteria will be certified as a sleep specialty. Independent Accreditation Committee was set up in TSSM for these accredited procedures. TSSM wishes to train more sleep physicians and technicians to achieve advanced technical trouble shooting capability and clinical sleep medicine related diagnosis and management in the future.

### Education

Sleep medicine began to be a medical course in the university since 2003 in Chang Gung University. Ning-Hung Chen started to set a sleep course majorly in clinical sleep medicine and PSG technique for the respiratory therapists. Later, sleep medicine became an essential course in respiratory therapy department in all universities. Dr. Chien-Min Yang and Dr. Lin-Lin Tsai, who are PhDs in Psychology, also started their general sleep course in psychologic school of the Fu-Jen University and National Chung Cheng Universities in 2003. We expect sleep medicine curriculum could be part of regular university courses in the future, and more sleep specialists and researchers could be inspired through the course. A survey of current sleep curriculum status of all Taiwan universities is shown in Table 3. Most sleep medicine related courses currently belong to elective courses of all universities, and around 18 % of all universities in Taiwan have these kinds of courses. In the future, TSSM will cooperate with those universities to recognize these sleep medicine related courses as the accreditation CMEs to encourage sleep medicine education in Taiwan.

**Table 3:** Sleep medicine related education course in the school of Taiwan

	Sleep medicine-related courses in university					
	School	Department	Obligatory/elective	Credit	Total number of schools	Percentage of all universities in TW
Medical laboratory science-related	–	–	–	–	–	
Nursing-related	–	–	–	–	–	
	Chang Gung University	Department of Respiratory Therapy	E	2		
Respiratory therapy-related	China Medical University	Department of Respiratory Therapy	E	2	3	
	Koahsiung Medical University	Department of Respiratory Therapy	O	2		
	National Taiwan University	Department of Psychology	E	3		18%
Psychology-related	Fu Jen Catholic University	Department of Clinical Psychology	E	2	4	
	National Chung Cheng University	Department of Psychology	E	2		
	Koahsiung Medical University	Department of Psychology	E	3		
Others	National Taiwan University	Department of Entomology	E	3	1	

*E* elective course, *O* obligatory course, *Credit* every credit means one hour lecture in a week

### The prevalence study by the Taiwan Society of Sleep Medicine

In 2006, an epidemiology study sponsored by Astellas Company investigated the epidemiology of sleep disorders in Taiwan. This study used computer-assisted telephone interviewing (CATI), a method using man-made telephone interviews assisted by a computer. Subjects aged over 15 living in Taiwan (including Taiwan island, Penghu County, Kinmen County and Lienchiang County) were investigation targets. The number of subjects to be interviewed was calculated according to the population distributions in each county and an estimated SDB prevalence (3–4 %). From 25 Oct 2006, to 6 Nov 2006, 11,649 individuals chosen randomly were interviewed by 40 well-trained telephone investigators. In total, 3862 (33.2 %) refused to be interviewed for any reason, and 3776 (32.4 %) persons did not complete the interview due to problems such as language barriers and poor telephone/cell phone quality. In total, 4011 (34.4 %) persons successfully completed the interview during the investigation period. This number reached the requirement of a 95 % confidence interval and bias of 3 %. In this study, we found the prevalence of insomnia is 24.8 %: 20.5 % in male and 29.3 % in female, *p* < 0.001. The prevalence of chronic insomnia (symptoms longer than 1 month) is 11.5 %: 9.9 % in male and 13.2 % in female, *p* < 0.01. The prevalence of snoring in these Taiwanese individuals was 51.9 % [95 % confidence interval (CI) 51.13–52.67 %], 60.8 % (95 % CI 58.67–62.93 %) in males and 42.5 % (95 % CI 40.26–44.74 %) in females. The prevalence of witnessed apnea during sleep was 2.6 % (95 % CI 2.1–3.1 %), 3.4 % (95 % CI 2.6–4.2 %) in males and 1.9 % (95 % CI 1.28–2.52 %) in females. The prevalence of snoring and witnessed apnea was significantly higher in males than in females (*p* < 0.05). Prevalence of hypertension, cardiovascular disease, diabetes mellitus, arthritis and backache was higher in those who snored or had witnessed apnea than those without these symptoms (*p* < 0.05) [1]. The prevalence of RLS in Taiwanese adults is 1.57 %. No gender difference is found in patients of RLS but females with RLS have higher incidence of menopausal syndrome [2].

## Sleep research in Taiwan

### Introduction

In the International Classification of Sleep Diseases third edition (ICSD-3), sleep breathing disorders (SDB) are characterized as four types including obstructive sleep apnea (OSA), central sleep apnea disorders, sleep-related hypoventilation disorders, and sleep-related hypoxemia disorder. In 1976, Guilleminault [3] first reported that OSA was manifested in recurrent airflow obstruction caused by a total (apnea) or partial collapse (hypopnea) of upper airway. OSA is one of the most common sleep breathing disorders affecting approximately 2 % of females and 4 % of males in the western countries [4]. In Asia, the estimated prevalence of OSA varied from 3.7 to 97.3 % [5].

Deficiency in medical resources associated with poverty leads to less efforts dedicated to the prevention, diagnosis and treatment of OSA for the Asian population in comparison with that in the developed countries. In consequence, the patients with untreated sleep apnea revealed a higher rate of cardiovascular and neuropsychologic morbidity [6, 7].

Given that Asia accounts for the largest proportion of population in the world, investigation on the OSA in Asian population is essential. The Japanese Society of Sleep Research (JSRS) first held a conference for sleep medicine in Tokyo in 1977. The Asian countries started up regional communities such as the Asian Sleep Research Society (ASRS) and participated in worldwide organizations such as the World Congress of Sleep Medicine (WASM) in the last two decades. Taiwan Society of Sleep Medicine (TSSM) was founded in 2002. In the study, we aimed to demonstrate the outcomes of OSA-related research in Asia. In particular, the progress driven by the studies in Taiwan will be discussed.

### Methodology for data collection

Data were obtained online from the Science Citation Index Expanded (SCI-EXPANDED) database of the Thomson Reuters’ Web of Science Core Collection (updated on 19 August 2015). Keywords including “apnea” and “hyponea” were used to search by applying the filters of the title and the publication years between 1991 and 2014. The type of document was restricted to an article. The results were further extracted subject to the field addresses of the Asian countries including Japan, China, Israel, Taiwan, South Korea, Hong Kong, India, Singapore, Saudi Arabia, Malaysia, Oman, Philippines, Indonesia, and Myanmar. The output was further refined to ensure that all country members of ASSM were included for data search.

### Results of sleep research in Taiwan

In total, 3668 articles were hit subject to the criteria for data search. By applying a filter, “front page” [8], articles exhibiting the keywords for search in the contents of front page such as title, abstract and authors were selected. The function of KeyWords Plus was used to explore the additional articles by examining the keywords in the cited bibliographies and footnotes in the ISI database (now Thomson Reuters, New York). Thus the title-word and author-keyword indices were significantly added [9]. The articles exclusively selected by the search of KeyWords Plus were not included. Finally, 2,623 OSA-related articles were selected for the involvement of member countries in ASSM (Asian Society of Sleep Medicine). The selected citations were exported from the Web of Science Core Collection by year and saved in Microsoft Excel 2010. The ID codes of the downloaded records were manually added [10]. The impact factors (IF) for each selected articles were referenced to the JCR (version 2014).

The articles reported from the addresses of England, Scotland, Northern Ireland, and Wales were stratified into a group of the United Kingdom (UK). The articles addressed to post-1997 Hong Kong were included under the heading of Hong Kong. In the format of Thompson Reuters Web of Science citation, the term of “corresponding author” usually shown on the articles is designated as “reprint author” [11]. The author of a single-author article was specified as the first author and the corresponding author [12]. Likewise, the single institute addressed in an article was characterized as the first author institute and the corresponding author institute. Contributions dedicated to the involved institutions or countries were estimated once they were affiliated in the author’s list. Six bibliometric indicators such as total articles, independent articles, collaborative articles, first-author articles, corresponding-author articles, and single-author articles were applied to grade the contributions of involved institutions or countries in an article [13].

**Fig. 1:**
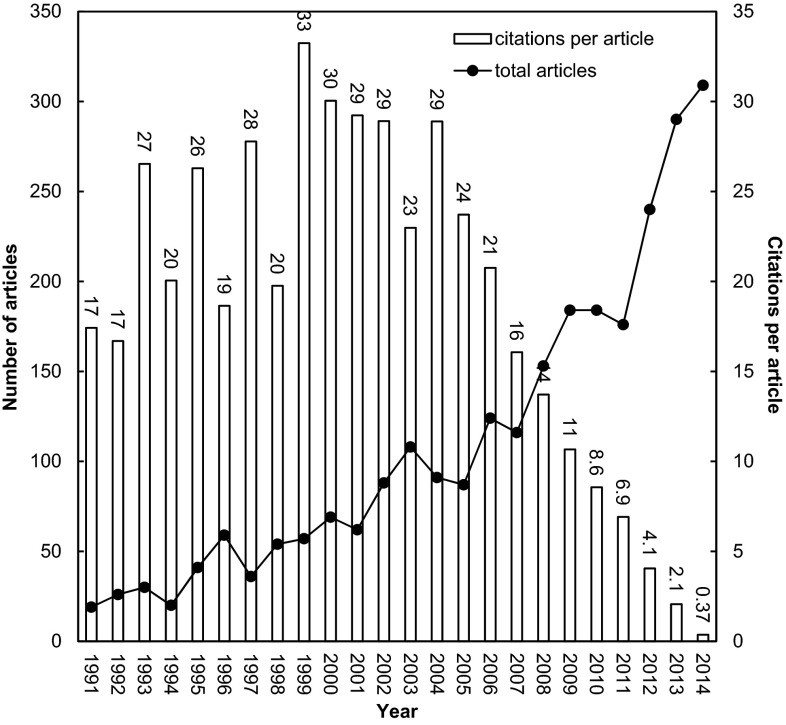
Trend of annual number of articles on obstructive sleep apnea during 1991–2014

Recently, the relationships between the numbers of frequently cited articles and their values of CPP (citations per publication = TC2014/publication) were analyzed by decade [12] and by year [11]. In Fig. 1, an overview of OSA-related publications involving the ASSM members is demonstrated in the annual number of articles and the CPP during 1991–2014.

**Fig. 2:**
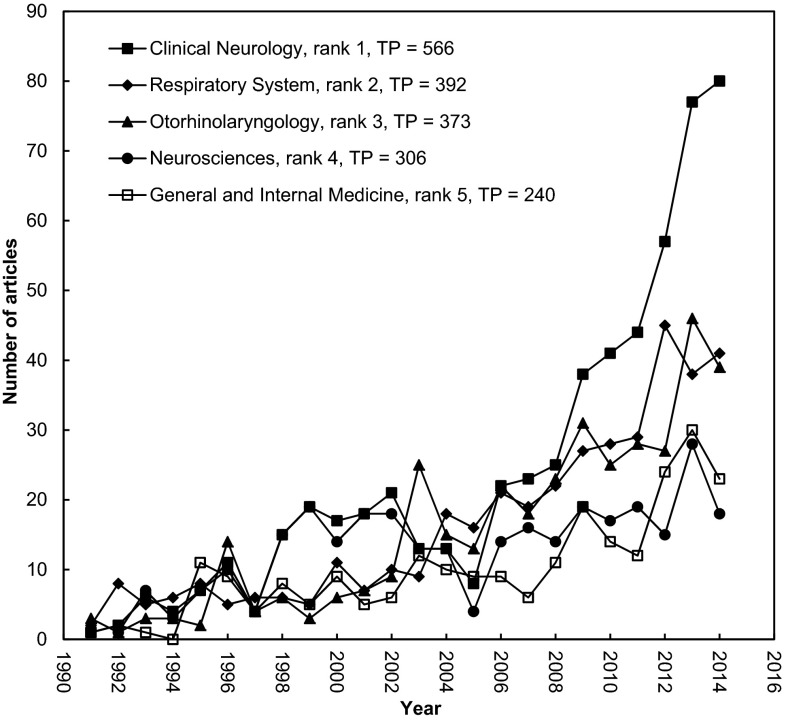
Publications of the top five productive (TP > 200) Web of Science categories during 1991–2014

It was found that the selected articles were most frequently published in the journals belonging to the field to clinical neurology followed by that of respiratory system (Fig. 2). Among the 2626 articles, the most frequently related topic was sleep and breathing (128, 4.95 %) and followed in order by topics of chest (94, 3.6 %), sleep (94, 3.6 %), sleep medicine (93, 3.5 %), psychiatry and clinical neurosciences (70, 2.7 %), acta oto-laryngologica (63, 2.4 %), traditional Chinese medical (59, 2.2 %), laryngoscope (59, 2.2 %), otolaryngology-head and neck surgery (58, 2.2 %), and internal medicine 54 (2.1 %) (Table 4).

**Table 4:** The 10 most productive journals (*TP* > 50) with the number of articles, impact factor, and category of journals during the period of 199q to 2014

Journal	*TP* (%)	*IF*_2014_	Web of Science category
Sleep and Breathing	128 (4.9)	2.482	Neurosciences and neurology respiratory system
Chest	94 (3.6)	7.483	General and internal medicine respiratory system
Sleep	94 (3.6)	4.591	Neurosciences and neurology
Sleep Medicine	93 (3.5)	3.154	Neurosciences and neurology
Psychiatry and Clinical Neurosciences	70 (2.7)	1.634	Neurosciences and neurology psychiatry
Acta Oto-Laryngologica	63 (2.4)	1.099	Otorhinolaryngology
Chinese Medical Journal	59 (2.2)	1.053	General and internal medicine
Laryngoscope	59 (2.2)	2.144	Research and experimental medicine otorhinolaryngology
Otolaryngology-Head and Neck Surgery	58 (2.2)	2.020	Otorhinolaryngology surgery
Internal Medicine	54 (2.1)	0.904	General and internal medicine

*TP* total number of articles,  *%* the percentage of articles of journals in total articles, *IF*_2014_ impact factor in 2014

In Asia, Japan is the country published the largest number of OSA‐related articles in terms of all six bibliometric indicators including total publications (TP), independent publications (IP), collaborative publications (CP), first‐author publications (FP), corresponding‐author publications (RP), and single‐author publications (SP) (Table 5). Meanwhile, China was ranked the second and Israel third. Taiwan was ranked the fourth in terms of the indicators including TP, FP, and RP whereas CR was the third and IP was the fifth. South Korea was fifth in the indicators of TP, CR, FP and RP but IP was ranked fourth (Table 5). The Asian institution ranked first in the number of OSA-related articles was Technion-Israel Institute of Technology and Tel Aviv University in Israel (Table 6). In Taiwan, Chang Gung Memorial Hospital and Chang Gung University were the fourth and the fifth, repectively. Both institutes were involved in 63 articles.

**Table 5:** Fourteen members of Asian Society of Sleep Medicine (ASSM)

Country	TP	*TP* *R* (%)	*IP* *R* (%)	*CP* *R* (%)	*FP* *R* (%)	*RP* *R* (%)	*SP* *R* (%)
Japan	992	1 (38)	1 (39)	1 (32)	1 (35)	1 (35)	1 (39)
China	433	2 (17)	2 (16)	2 (19)	2 (14)	2 (14)	7 (3.5)
Israel	305	3 (12)	3 (11)	4 (15)	3 (10)	3 (10)	2 (16)
Taiwan	295	4 (11)	5 (10)	3 (16)	4 (10)	4 (9.4)	5 (8.8)
South Korea	266	5 (10)	4 (11)	5 (8.3)	5 (9.1)	5 (9.2)	3 (12)
Hong Kong	113	6 (4.3)	6 (4.5)	7 (3.7)	6 (4.1)	6 (4.1)	7 (3.5)
India	106	7 (4.0)	7 (4.2)	8 (3.5)	7 (3.7)	7 (3.6)	6 (5.3)
Singapore	66	8 (2.5)	9 (1.6)	6 (6.2)	8 (2.1)	8 (2.0)	N/A
Saudi Arabia	53	9 (2.0)	8 (1.9)	9 (2.5)	9 (1.7)	9 (1.7)	3 (12)
Malaysia	19	10 (0.72)	10 (0.62)	10 (1.2)	10 (0.50)	10 (0.50)	N/A
Oman	11	11 (0.42)	11 (0.33)	11 (0.77)	11 (0.30)	11 (0.31)	N/A
Philippines	1	12 (0.038)	N/A	12 (0.19)	N/A	N/A	N/A
Indonesia	0	N/A	N/A	N/A	N/A	N/A	N/A
Myanmar	0	N/A	N/A	N/A	N/A	N/A	N/A

*TP* total number of articles, *TP R (%)* rank and the percentage of total articles, *IP R (%)* rank and the percentage of independent articles, *CP R (%)* rank and the percentage of international collaborative articles, *FP R (%)* rank and the percentage of first authored articles, *RP R (%)* rank and the percentage of the corresponding authored articles, *IP R* rank and the percentage of the single authored articles

**Table 6:** The top 10 most productive institutions

Institution	TP	*TPR* (%)	*SPR* (%)	*ICPR* (%)	*NCPR* (%)	*FPR* (%)	*RPR* (%)	*SPR* (%)
Technion-Israel Institute of Technology, Israel	100	1 (3.8)	1 (3.3)	3 (3.7)	4 (3.8)	2 (2.0)	2 (1.9)	3 (7.0)
Tel Aviv University, Israel	90	2 (3.4)	26 (0.76)	7 (1.7)	1 (6.7)	26 (0.69)	30 (0.58)	N/A
Seoul National University, South Korea	79	3 (3.0)	3 (2.7)	11 (1.2)	2 (4.3)	1 (2.2)	1 (2.4)	9 (1.8)
Chang Gung Memorial Hospital, Taiwan	63	4 (2.4)	24 (0.85)	2 (3.9)	8 (2.4)	7 (1.3)	11 (1.2)	N/A
Chang Gung University, Taiwan	63	4 (2.4)	40 (0.57)	1 (4.8)	12 (2.2)	20 (0.84)	25 (0.66)	N/A
Kyoto University, Japan	56	6 (2.1)	6 (2.5)	99 (0.19)	6 (2.5)	4 (1.8)	5 (1.7)	1 (14)
University of Hong Kong, Hong Kong	56	6 (2.1)	2 (3.2)	49 (0.39)	28 (1.3)	3 (1.8)	3 (1.9)	5 (3.5)
Chiba University, Japan	51	8 (1.9)	3 (2.7)	29 (0.58)	28 (1.3)	5 (1.7)	4 (1.7)	4 (5.3)
Nagoya University, Japan	51	8 (1.9)	76 (0.28)	15 (1.0)	3 (4.1)	14 (1.0)	14 (0.93)	9 (1.8)
Tohoku University, Japan	46	10 (1.8)	3 (2.7)	49 (0.39)	27 (1.4)	9 (1.2)	8 (1.2)	N/A

*TP* total number of articles, *TPR (%)* rank and the percentage of total articles, *SPR (%)* rank and the percentage of single institution articles, *ICPR (%)* rank and the percentage of internationally collaborative articles, *NCPR (%)* rank and the percentage of nationally collaborative articles, *FPR (%)* rank and the percentage of first author articles, *RPR (%)* rank and the percentage of the corresponding authored articles, *SPR (%)* rank and the percentage of single author articles, *N/A* not available

### Discussion about the sleep research in Taiwan

The quantity of OSA-related articles published with the involvement of ASSM member countries only contributed 17 % of the global publications in the same field (2632 vs. 16,267). In the last two decades, the number of OSA-related articles increased quickly and article citations peaked in 1999 (Fig. 1). Professional institutions such as JJSRS, ASRS and WASM exhibited critical roles on accelerating the growth of research outcomes.

The Asian country-contributed research articles related to OSA were most frequently published in the journal category of clinical neurology science, followed by that of respiratory system (Fig. 2). In particular, clinical neurology-related articles increased dramatically in quantity since 2006 when compared to the fields of respiratory system and otorhinolaryngology. The journals related to sleep and breathing were found to be more accessible for publishing the OSA-related researches in the perspective of clinical neurology. More studies conducted in Asia have demonstrated that OSA correlated with neurologic disorders.

**Fig. 3:**
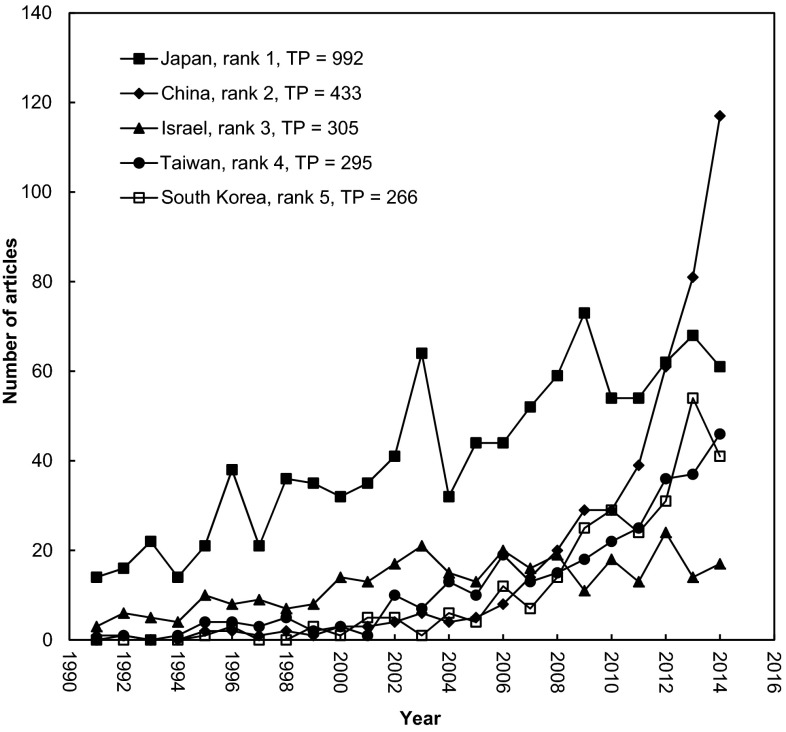
Publications of the five most productive countries during 1991–2014

Among the members of ASSM, Japan contributed the most publication of articles. Since 1994, the growth rate in quantity was persistent but revealing a trend in decline (Fig. 3). China showed a rapid increase in the number of articles since 2011 and ranked second, followed by Israel. Taiwan and Korea performed similarly in OAS-related research since 2002, the year of TSSM.

**Fig. 4:**
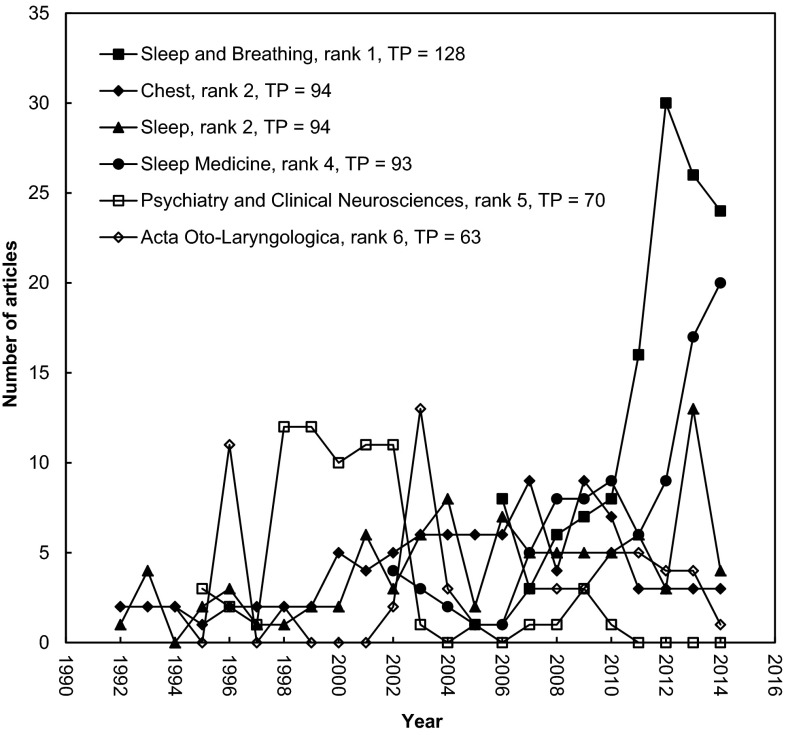
Publications of the top six productive (TP > 60) journals during 1991–2014

The Asian institution most frequently affiliated in the involvement of OSA-related articles was Technion-Israel Institute of Technology and Tel Aviv University in Israel (Table 6). Since 2010, Asia country-contributed research articles related to OSA were most frequently published in sleep and breathing and chest (Fig. 4). Following the Annual Sleep Apnea Conference 2006 held in Korea, Seoul Nation University showed significant improvement in performance. In Taiwan, Chang Gung Memorial Hospital and Chang Gung University were ranked fourth and fifth, respectively, in terms of quantity of OSA-related articles with Asian involvement. Both institutions were involved in 63 articles. In fact, the hospital and university entitled Chang Gung are private institutions belonging to a Taiwanese conglomerate. Under the circumstances assuming the fourth and fifth as a single institution, Chang Gung became the topmost in the performance of publishing OSA-related research articles.

### Conclusions about the sleep research in Taiwan

The research articles for OSA based on the key word “apnea” and “hyponea” from the ASSM accounted for 17 %. The most productive institution was Technion-Israel Institute of Technology and, Tel Aviv University in Israel. Significant increase in the number of reports from Korea was observed after the Annual Sleep Apnea Conference hold in Korea since 2006. In Taiwan, Chang Gung Memorial Hospital and Chang Gung University were the fourth and fifth productive institutions that published 63 and 63 articles each. Because there is a strong connection between the two institutions, the papers together enable “Chang Gung” to be the most productive institution in the field of obstructive sleep apnea.

In Asia, Japan leads in the quantity of publication and the Japanese research institutes performed evenly. China revealed rapid growth in the number of articles since 2011. Taiwan and Korea are competing to enhance their involvement in the OSA-related research. The academic organizations such as JJSRS, ASRS and WASM play an important role on promoting the research capacity. In Taiwan, Chang Gung Memorial Hospital with Chang Gung University was a representative example of successfully integrating the resources of basic and clinical research. It is not unusual in Asia. ASSM exhibits a critical role on enhancing the outcomes of research related to the sleep disorders.

### The future

The sleep medicine in Taiwan developed rapidly and smoothly by the efforts of pioneers in this field. There are several problems for the future development that the sleep specialists and the society will encounter.

#### Insurance issue

The PSG study is covered by national insurance in Taiwan and that promoted the development of sleep study in the past decades. However, the global budget and capitals of the health care in Taiwan limit the expansion of sleep study. Compared to the increasing number of patients of sleep disorders, the service of sleep beds is far below the need for the 23 million population in Taiwan. The reimbursement of sleep study, technicians and physicians are so very low that the physicians cannot offer the salary from the practice of sleep medicine. These problems will hinder the development of sleep medicine in Taiwan.

#### Profession affiliation

Knowledge of sleep medicine is important for specialists that take care of the patients with sleep disorders. Understanding the knowledge of sleep medicine for different disciplinaries prevent malpractice of the treatment for patients. That is also the reason why sleep medicine has become a specialty. However, the reimbursement of sleep study in USA is high enough to support the physicians to practice sleep medicine alone but not in other countries. Most of the physicians in Taiwan have to take care of patients other than sleep disorders in their original specialty, e.g. the pulmonologist have to take care of asthma, lung cancer even critical patients in ICU as well as patients with sleep complaints. The reimbursement in health care system limits sleep physicians’ focus on the practice of sleep medicine. As a result, sleep medicine cannot be an independent department on the health care system in Taiwan. As the newly developed medical science, what is the better model for further development of sleep medicine in Taiwan is also the issue that sleep physicians have to think it over.

#### Research

The research promotes the knowledge of medicine and improves clinical practice. It is more than important to emphasize the research of sleep medicine in every disciplinary. The research and publication of sleep medicine in Taiwan are still young. It is the responsibility for sleep specialists and the society to promote sleep research in Taiwan.

#### Education

As shown previously, education of sleep medicine in Taiwan is the focus of technicians and psychologists. Sleep medicine is a basic medicine that every medical student should understand. The medical course of sleep medicine at the university should be expanded to all departments to become a basic medical science.

## Conclusion

Although the development of sleep medicine in Taiwan is smooth and rapid on the past decades, it is still very young in the field of medicine and needs much more effort to consolidate the achievement. The society of sleep medicine in Taiwan has the responsibility to discuss and take action for these issues.
